# Flammability of Polymer Compositions Filled with Wheat Bran

**DOI:** 10.3390/ma15248955

**Published:** 2022-12-15

**Authors:** Emil Sasimowski, Bronisław Samujło, Marta Grochowicz, Łukasz Majewski

**Affiliations:** 1 Department of Technology and Polymer Processing, Faculty of Mechanical Engineering, Lublin University of Technology, 20-618 Lublin, Poland; 2 Department of Polymer Chemistry, Institute of Chemical Sciences, Faculty of Chemistry, Maria Curie-Sklodowska University, 20-614 Lublin, Poland

**Keywords:** composite, flammability, biofiller, agro-waste materials, agro-flour filler, natural filler, lignocellulosic materials, biopolymer

## Abstract

The article presents the results of flammability tests on polymer compositions with wheat bran (WB) as the applied filler, and polyethylene (PE) or poly(butylene succinate) (PBS) as the matrix material. Tests were conducted using samples of compositions containing 10, 30 and 50%wt wheat bran. The test samples were manufactured by injection moulding from compositions previously produced by extrusion pelleting. For comparative purposes, samples made only of the plastics used for the composition matrix were also examined. Flammability tests were carried out in accordance with the recommendations of EN 60695-11-10 Part 11–10 with horizontal and vertical positioning of the sample, using a universal flammability-test-stand. During the flammability tests, changes in the temperature field in the area of the burning sample were also recorded, using a thermal imaging camera. Sample residues after flammability tests were also examined with infrared spectroscopy (FTIR) to assess their thermal destruction. The results of the study showed a significant increase in flammability with bran content for both PE and PBS matrix compositions. Clear differences were also found in the combustion behaviour of the matrix materials alone. Both the burning rate and maximum flame temperature were lower in favour of PBS. PBS compositions with wheat bran also showed lower flammability, compared with their PE matrix counterparts.

## 1. Introduction

The increase in demand for relatively inexpensive polymeric materials that meet environmental requirements, which has been observed for several years, has led to great interest in polymer compositions containing fillers of natural origin. These compositions, most of which degrade under environmental conditions, are expected to replace or at least partially reduce the use of traditional petrochemical plastics [1,2,3,4]. One direction of ongoing work on these environmentally friendly materials is the use of plant-based ingredients. Their most common sources are plant fragments that constitute process waste from agriculture, food, wood or textile industries, which act as fillers for polymer compositions [2,3,5,6,7,8]. These include rice bran and hulls [9,10,11,12], wheat bran [13,14,15,16,17], shells from various nut species [18,19,20,21,22,23,24], dust from various tree species [8,25,26], residues from palm oil production [27] and sugarcane (bagasse) processing [28], wheat straw [29], sunflower and corn stalks [30], and barley hulls [31]. Such fillers, whose main components are cellulose, hemicellulose and lignin, are called lignocellulosic fillers [13,25]. The matrix of the composition, however, is made up of biodegradable or petrochemical polymers [32]. The main purpose of using such compositions is usually to significantly reduce the use of petrochemical polymers and expensive biodegradable polymers. Nowadays, it is also of great importance to achieve biodegradability or shorten the biodegradation time of post-consumer products by using a high filling level [16,33,34,35,36,37]. Biodegradable polymers such as poly(butylene succinate), polycaprolactone or polylactide can be used for a long time under standard conditions, and only biodegrade when subjected to industrial composting [38,39,40].

The scope of application of polymer compositions containing fillers of natural origin is becoming wider and wider, and is not limited only to products of common use. Increasingly, these types of materials find technical applications where low flammability or even ‘non-flammability’ is required, including automotive, home furnishings and woodwork. The introduction of natural fillers generally limits the maximum processing temperature of the composition to approximately 150 °C, which results in selecting as its matrix thermoplastic polymers such as polyethylene, polystyrene, polypropylene, as well as biodegradable polymers such as poly(butylene succinate), which, in addition to low processing temperature, also boast high flammability. The introduction of natural fillers into polymer compositions often increases flammability [41,42]. Therefore, in addition to studying the processability and physical properties of the compositions, it is equally important to know their characteristics, including flammability. Learning about the effect on flammability of the use of natural fillers, especially for biodegradable plastics, would enable their appropriate modification in terms of increasing ignition resistance, reducing the rate of heat release, smoke generation and the rate of flame spread. This could help reduce the fire hazard they pose.

This article is a continuation of a comprehensive study of the properties of polymer biocomposites containing wheat bran. Studies of the physical and processing properties of the compositions carried out so far by the authors of this article have shown that the bran content has a significant effect on these properties [14,15,16,17,43,44]. The purpose of the presented study was to determine the effect of using a filler in the form of wheat bran on the flammability of the obtained polymer biocomposition. Both the effect of the mass content of wheat bran in the composition samples in the range of 10–50%wt, as well as the matrix material—polyethylene (PE) or poly(butylene succinate) (PBS)—were investigated. The ATR–FTIR method was applied in the research carried out on the residual samples after the flammability test, to evaluate their thermal destruction.

## 2. Experiments

### 2.1. Materials

The matrix materials in the biocomposites tested were polyethylene and poly(butylene succinate). Dowlex 2631.10UE polyethylene [45], manufactured by The DOW Chemical Company, was used to produce the biocomposition. It is designed for injection moulding of parts with high dimensional accuracy, and for rotary casting of thin-walled parts. The PBS used for the purposes of the study was BioPBS FZ91 PB [46], produced by PTT MCC BIOCHEM CO., LTD, Bangkok, Thailand. The material is designed for the manufacture of general-purpose products by injection moulding. The tested biocomposition samples were produced using wheat bran (WB) filler from a local mill near the city of Lublin (Poland). Bran, or grain husks in the form of thin flakes, was a byproduct of milling wheat into flour.

Test samples of the polymer biocomposites under study were obtained using a screw injection moulding machine from composite pellets previously produced by extrusion. The preparation of the composition components, the use of the device, and the conditions of the extrusion and injection-moulding process have been described in detail in previous works [14,15,16,43,44].

### 2.2. Research Programme and Methodology

Experimental tests were carried out according to the adopted design of experiment (DOE), the experimental layouts of which are shown in Table 1. The variable factors assumed for the study were the mass proportion of wheat bran, u = 0–50%wt, in the biocomposition, and the type of matrix material—polyethylene (PE) or poly(butylene succinate) (PBS).

Flammability tests were carried out according to the recommendations of PN-EN 60695-11-10 with horizontal and vertical sample positioning, using a universal-material flammability test stand, which is part of the equipment of the Department of Technology and Polymer Processing of Lublin University of Technology. Five specimens were used for each material sample in the horizontal and in the vertical burning tests. The ignition source was a 50 W propane-fuelled gas burner. Measurements of the temperature-field changes in the area of the burning sample were carried out using the ER005-25 V-20 thermal imaging camera (Vigo System S.A.). The infrared detection system of the V-20 camera allowed temperature measurements to be made in the range of 15 to 250 °C. Thermal images were recorded from a distance of 700 mm, and the time of generating one scan was 15 s. Analysis of the recorded thermal images (thermograms) was carried out using special ThermV-20 software from Vigo S.A.

In the flammability test performed using Method A, the sample was fixed horizontally in the holder. Keeping the centre axis of the burner tube inclined at 45° to the horizontal plane, the burner flame was applied to the lower edge of the free end of the sample in such a way that the flame encompassed it for approximately 6 mm. The test flame was held for 30 ± 1 s without changing its position, or the burner was moved away when the flame front on the sample reached the 25 mm mark. When the flame front reached the 25 mm mark a timer was activated and the time after which the flame front reached the 100 mm mark was recorded. For each sample, the linear burning rate *v* was calculated in accordance with the relation

$$v=\frac{60\cdot L}{t}\left[\frac{\mathrm{mm}}{\min}\right]$$

in which *v* is the linear burning rate in millimetres per minute, *L* is the length of sample destruction in millimetres, and *t* is the time in seconds. The material was classified in accordance with the criteria given in the recommendations of EN 60695-11-10:2014. The standard specifies a higher and a lower flammability class. A higher flammability class, denoted as HB40, is assigned when the sample does not ignite, the flame front travels less than 100 mm, or the linear burning rate is less than 40 mm/min. The lower flammability class HB75 is assigned when the linear burning rate does not exceed 75 mm/min and the flame front travels a distance greater than 100 mm.

In the vertical burning test (Method B), after the sample was fixed vertically, the burner was set in such a way so that it was possible to apply a flame covering the end of the sample at 10 mm length. Due to falling drops of molten or burning material, as per recommendations of the cited standard, the burner was set at an angle of 45° while maintaining the indicated distance of the flame centre from the end of the sample. The flame was held in position for 10 s, then moved away from the sample. In the vertical burning test, the standard specifies three classes of flammability. The highest flammability class, V-0, is assigned when the burning time of a single sample does not exceed 10 s, the total burning time of 5 samples is shorter than 50 s, there is no dripping of the burning sample and no ignition of the cotton batting placed under the sample clamp. The lower flammability class of the material, with the symbol V-1, is assigned when the burning time of a single sample is shorter than 30 s, the total burning time of 5 samples does not exceed 250 s, there is no dripping of the burning sample, and no ignition of the cotton batting. The lowest flammability class, V-2, is assigned when the burning time of a single sample is shorter than 30 s and the total burning time of 5 samples does not exceed 250 s but there is dripping of the burning sample, and ignition of the cotton batting occurs.

Each time, the burning time of the sample was measured after the test flame was moved away, and in almost all the cases tested (except for samples PBS10, PBS30) the flame covered the entire length of the sample within a dozen to tens of seconds, and it was impossible to precisely measure the burning and glowing time, and therefore to determine the flammability class in the sample. A thermal image of the burning sample was recorded each time, immediately after the burner flame was removed.

The sample residues after the flammability test were tested using the ATR–FTIR method. The infrared spectra (FTIR) of tested samples were taken using a Tensor 27 spectrometer (Bruker, Germany) equipped with an ATR (attenuated total reflectance) module with diamond crystal. The spectra were recorded from 600 to 4000 cm^−1^ with 32 scans per spectrum and a resolution of 4 cm^−1^.

## 3. Results

### 3.1. Horizontal Flammability Tests

Table 2 shows the results of the horizontal flammability tests. In the case of polyethylene (PE), the introduction of bran into the composition resulted in an increase in the linear burning rate and, in the case of sample PE30, a deterioration of the flammability class to HB75. This may be due to the decrease in the enthalpy of melting of the material with the increase in the filler content [44]. As a consequence, there was more intense dripping of the burning material, and an increase in the burning rate. In the case of 50%wt content of the above ingredients, a favourable increase in burning time and a decrease in burning rate were observed, which is characteristic of highly-filled polyolefin plastics [47,48]. The high content of the filler decelerates the dripping of the burning material, thus slowing down the combustion process. In the case of the second tested material (PBS), the linear burning rate was significantly lower than that obtained for polyethylene, but the introduction of a combustible filler caused an increase in burning-rate value. In this case, a similar effect as that in the case of PE was observed to reduce the linear burning rate at the filler content of 50%wt, in PBS. 

### 3.2. Vertical Flammability Tests

The results of the vertical flammability tests are shown in Table 3. For all tested materials, except PBS10 and PBS30, the samples burned completely, ignition of the cotton indicator occurred, and the flame covered the entire sample. After a few tens of seconds, the flame front reached the upper clip of the holder. The approximate burning time given in Table 2 was recorded from the moment of moving the burner away, to the flame front reaching the upper clip of the holder. 

The results of the measurements make it impossible to classify the samples into V0, V1 or V2 flammability classes. Unlike the results of the horizontal test, the addition of filler in PBS increased the burning time, but increasing the filler contents to 30 and 50% resulted in complete combustion of the sample. In the case of the PBS and PBS10 samples, classification in the V-2 flammability class was impossible, due only to the burning time exceeding 250 s for the set of five samples, which was too long according to the requirements of the cited standard, but in these cases the samples were extinguished before the flame reached the holder.

### 3.3. Sample Temperature during Horizontal Combustion

**Figure 1:**
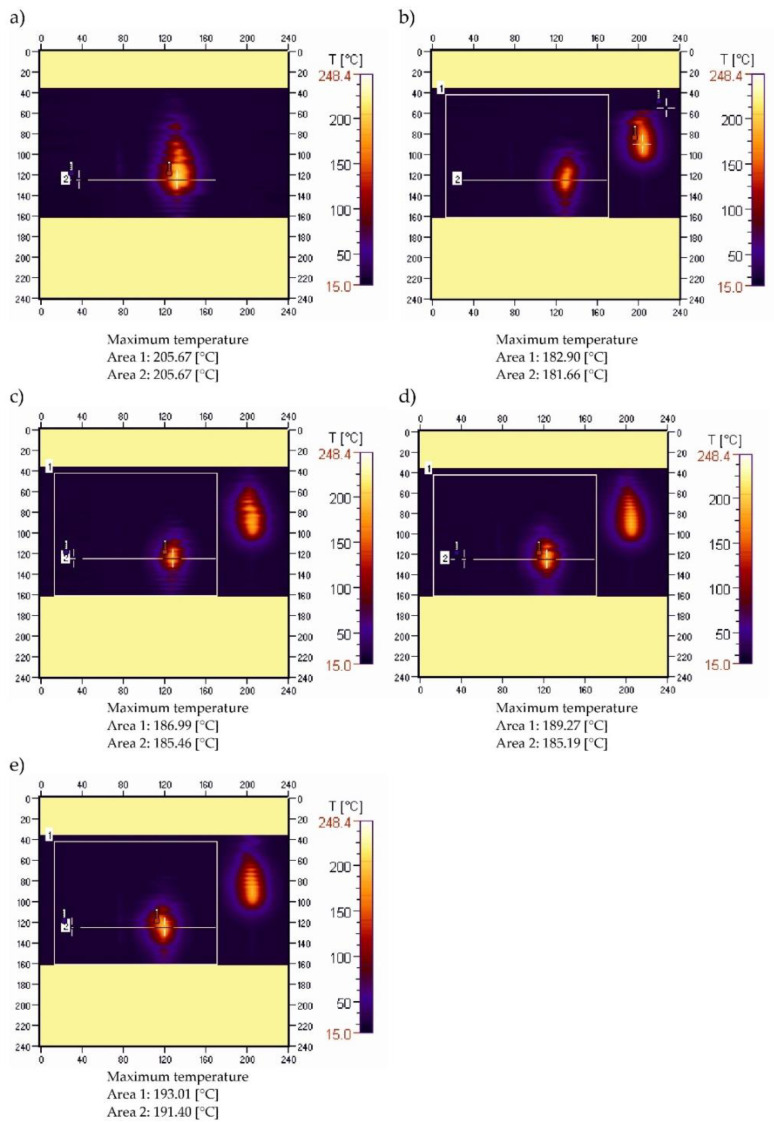
Thermograms of pure PE sample recorded during ignition (**a**) and after: (**b**)—15, (**c**)—30, (**d**)—45, (**e**)—60 s after the ignition source was removed.

Analysis of the temperature field in the area of the burning sample in the horizontal combustion test involves determining the changes in the maximum temperature in the area marked 1 in Figure 1. 

Thermal images were recorded during ignition at 15, 30, 45 and 60 s after the ignition source was moved away. The temperature changes recorded along a straight line running through the longitudinal axis of the sample were also analysed (Figure 1, area 2). Example thermal images for an unmodified PE sample are shown in Figure 1. 

**Figure 2:**
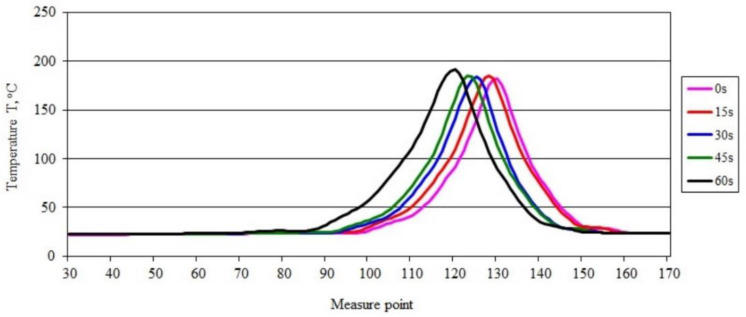
Temperature along the burning PE sample recorded in area 2.

The maximum temperature in the burning area of the sample (Figure 1a–e), which was 205.67 °C during ignition, initially decreased to 182.90 °C, and then increased until the last measurement taken 60 s after the ignition source was removed, when it reached 193.01 °C. Thus, the increase in the temperature of the burning sample is relatively small, amounting to just over 5.5% compared with the value recorded immediately after removal of the ignition source, being concurrently indicative of gradual flame development. This is reflected in the course of changes in the temperature profile along the sample axis (Figure 2). Figure 2 also shows a gradual increase in the value of the maximum temperature in the axis of the sample with the burning time, from a value of 181.66 °C to 191.40 °C after 60 s (0.16 °C /s) from the removal of the burner flame. Shifting of the peak recorded temperature-values along the sample emphasizes the flame travel as combustion proceeds.

**Figure 3:**
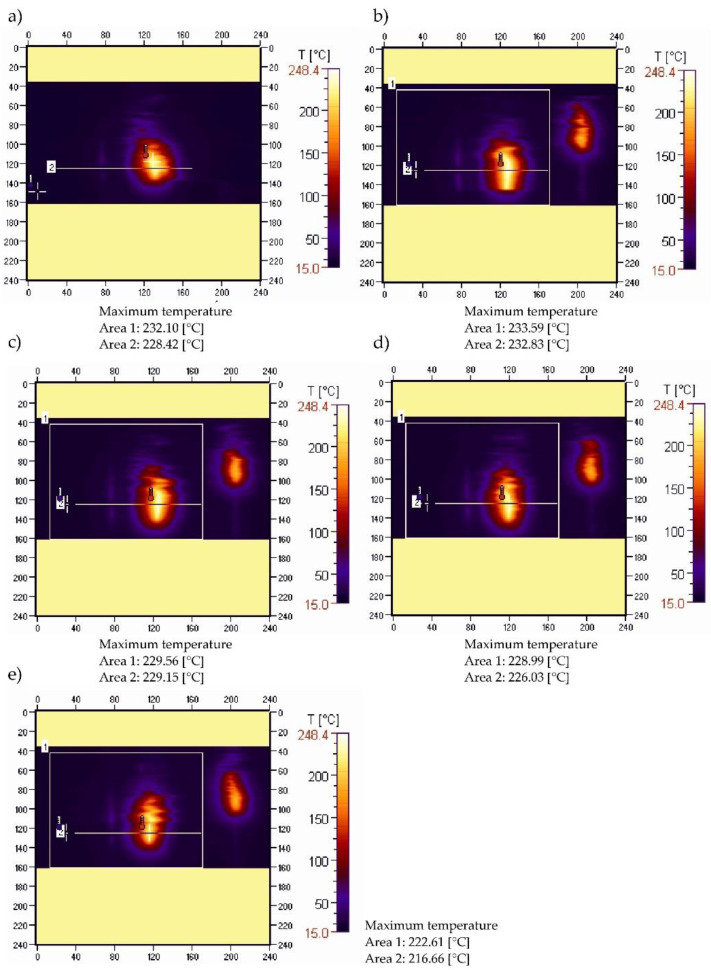
Thermograms of PE50 sample recorded during ignition (**a**) and after: (**b**)—15, (**c**)—30, (**d**)—45, (**e**)—60 s after the ignition source was removed.

Figure 3 shows thermal images recorded during the ignition and combustion test of sample PE50.

**Figure 4:**
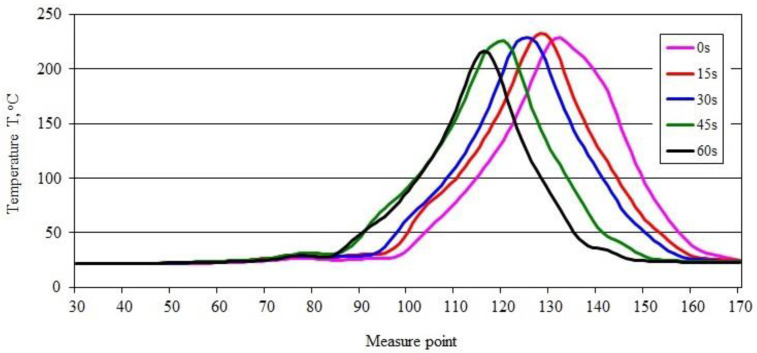
Temperature along the burning PE50 sample recorded in area 2.

A slightly different pattern of maximum temperature changes in the burning area was obtained for the PE50 sample (Figure 3a–e). The temperature value, after an initial rise from 232.10 °C to 233.59 °C (Figure 3a,b), gradually decreased to 222.61 °C (Figure 3e), which means a more than 4.7% relative temperature drop in the burning area within 45 s. This indicates a slight decrease in the intensity of the combustion process over time. These changes are confirmed by the course of the recorded temperature curves along the sample (Figure 4). Based on the course of their changes over time, after an initial slight increase (of approximately 4 °C), a gradual decrease can be observed in the value of the maximum temperature in the axis of the sample, with the passage of burning time. Its value after 15 s of 232.83 °C decreased to 216.66 °C (0.36 °C/s), 60 s after moving the burner flame away (Figure 4). Shifting of the peak recorded temperature-values along the sample emphasizes the flame travel as combustion proceeds.

**Figure 5:**
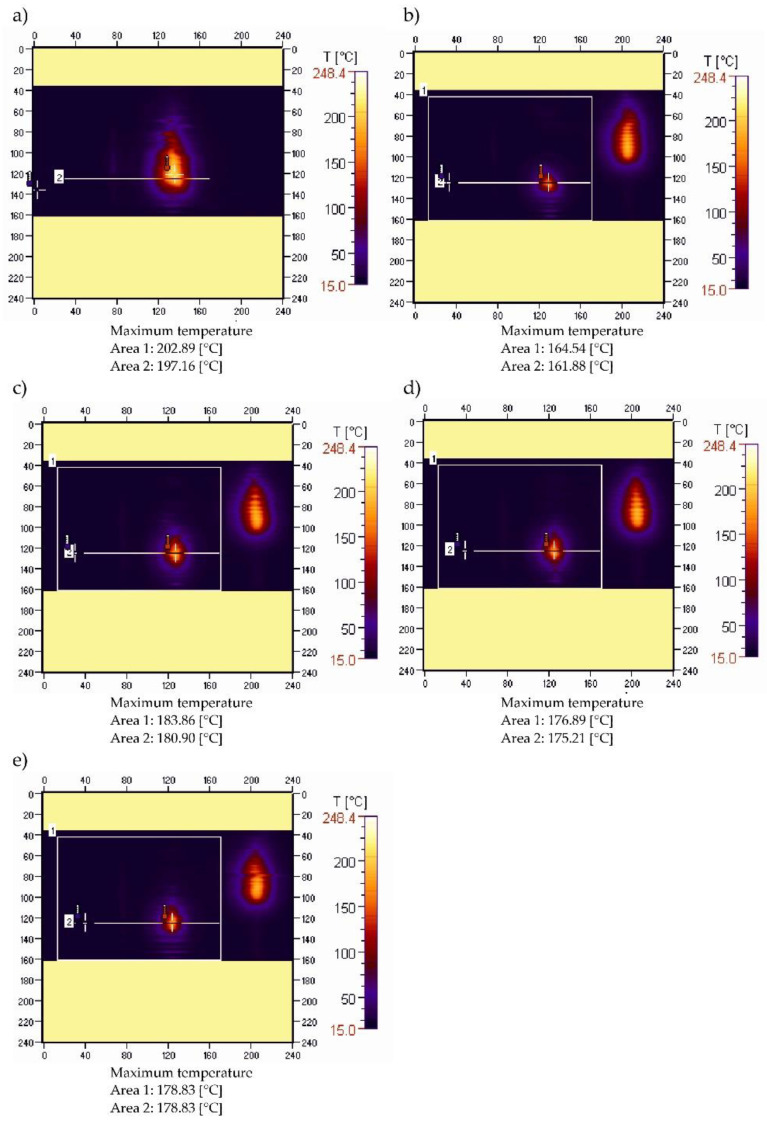
Thermograms of PBS sample recorded during ignition (**a**) and after: (**b**)—15, (**c**)—30, (**d**)—45, (**e**)—60 s after the ignition source was removed.

**Figure 6:**
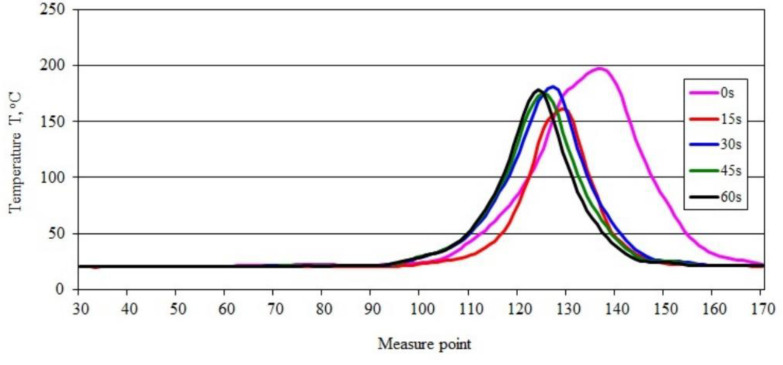
Temperature along the burning PBS sample recorded in area 2.

Figure 5 shows thermal images recorded during the ignition and combustion test of sample PBS without filler. A slightly different course of temperature changes in the burning area was obtained (Figure 5a–e). The temperature value, after initially dropping from 202.89 °C to 164.54 °C (Figure 5a,b), increased to 183.66 °C and then slightly decreased to 178.83 °C (Figure 5e). Within 60 s, the maximum temperature in the burning area was reduced by slightly more than 24 °C, but the initial significant reduction in flame was followed by its stabilization and burning of the sample over the entire test section. These changes are confirmed by the course of the recorded temperature curves along the sample (Figure 6).

The curves of temperature changes over time along the sample indicate that the initial decrease (of approximately 24 °C) was followed by a slight increase in temperature, and its stabilization (Figure 6). The shift the peak of the recorded temperature values along the sample reveals an initially intense flame travel, followed by a significant reduction in the speed of its movement.

**Figure 7:**
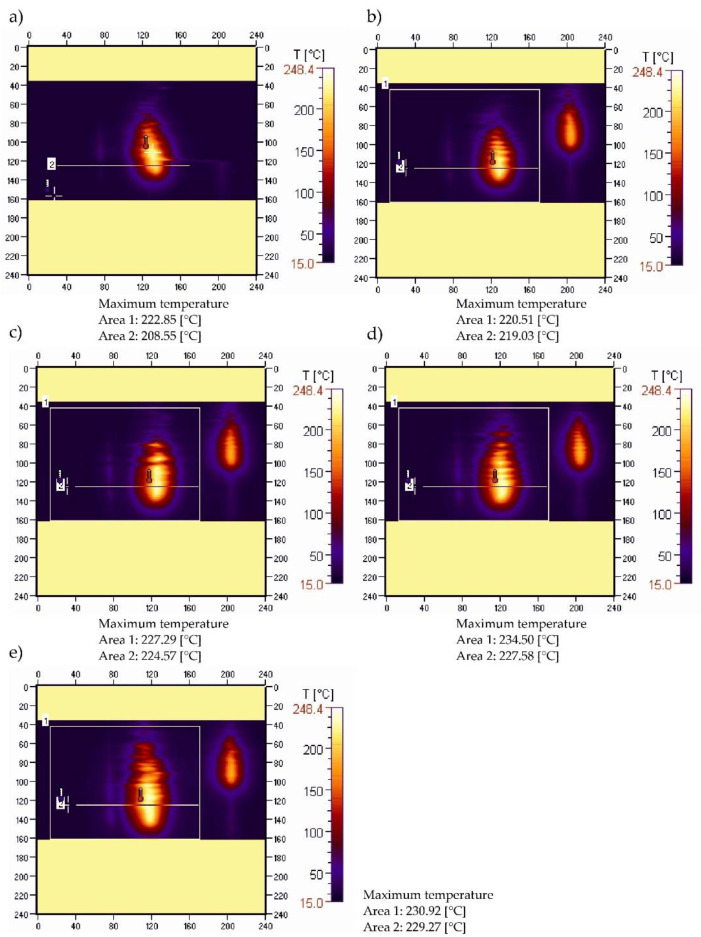
Thermograms of PBS50 sample recorded during ignition (**a**), and after: (**b**)—15, (**c**)—30, (**d**)—45, (**e**)—60 s after the ignition source was removed.

Figure 7 shows thermal images recorded during the ignition and combustion test of sample PBS50.

**Figure 8:**
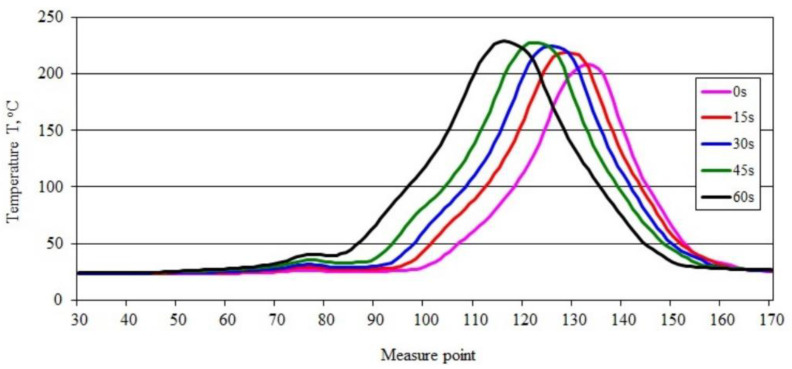
Temperature along the burning PBS50 sample recorded in area 2.

The course of changes in the maximum temperature in the burning area obtained for the PBS50 sample shown in Figure 7a–e, highlights the development of the flame during the burning of the sample. The value of the maximum temperature in the burning area, after an initial slight decrease from 222.85 °C to 220.51 °C, increased to 234.50 °C after 45 s and decreased slightly to 230.92 °C after 60 s (Figure 7e). Within 45 s, the maximum temperature in the burning area increased by almost 12 °C. These changes are confirmed by the course of the recorded temperature-change-curves along the sample axis (Figure 8).

The curves of maximum temperature changes over time along the sample indicate that there was a steady increase in temperature from 208.55 to 229.27 °C (Figure 8). The shift of the peak of the recorded temperature values along the sample reveals an intense flame travel along the sample.

**Figure 9:**
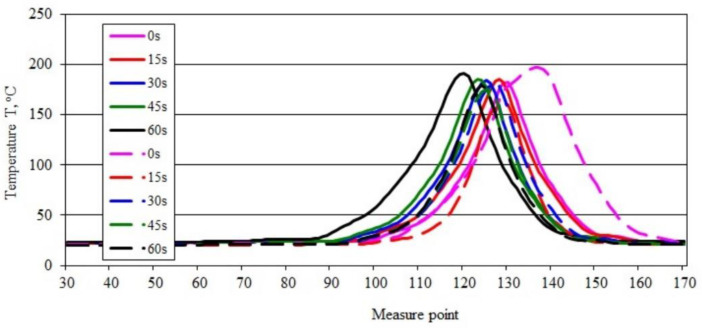
Temperature along the burning PE and PBS sample recorded in area 2 (description in the text).

For comparison purposes, Figure 9 shows a graph of the temperature change along a burning PE (solid lines) and PBS (dashed lines) sample recorded in area 2. The position of the curves on the graph indicates a lower rate of flame travel and a lower maximum temperature in the longitudinal axis of the samples, when burning PBS. 

**Figure 10:**
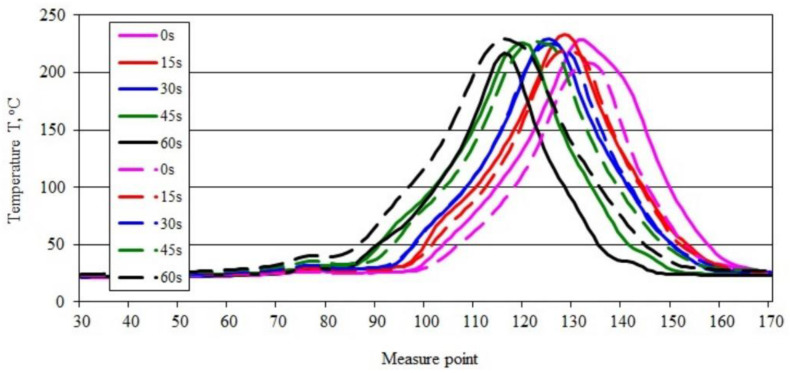
Temperature along the burning PE50 PBS50 sample recorded in area 2 (description in the text).

For both the tested materials with 50% filler content, the results were different from those obtained for unfilled materials (Figure 10). The curves of temperature change over time for PE50 testify to a gradual decrease in the intensity of the combustion process, which is also indicated by a decrease in the maximum flame temperature at the sample axis. The curves obtained for PBS50, on the other hand, show a steady increase in the maximum temperature in the longitudinal axis of the samples, indicating flame propagation. 

### 3.4. Sample Temperature during Vertical Combustion 

**Figure 11:**
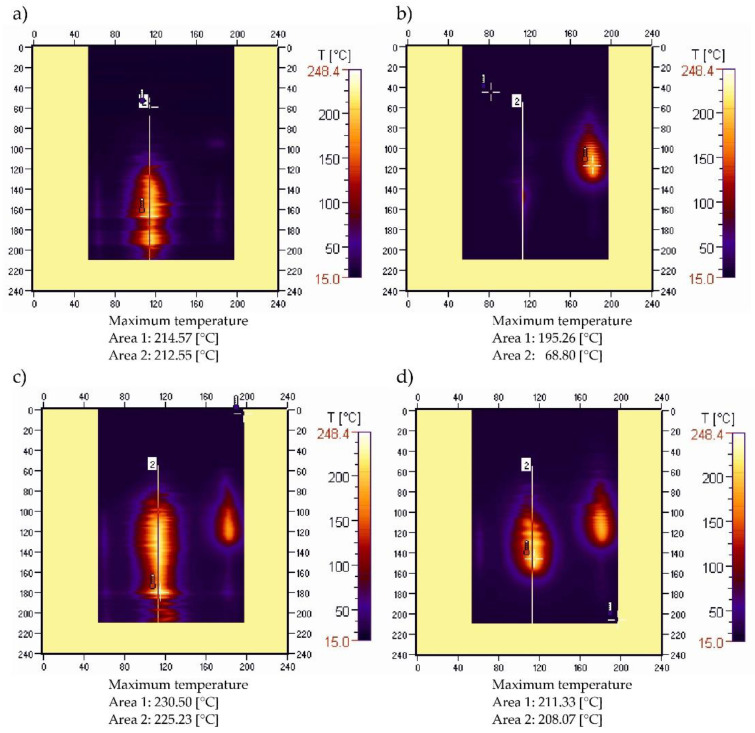
Thermograms of unmodified PE (**a**), PBS (**b**), PE (**c**), and PBS (**d**) samples, containing 50% filler content.

Examples of recorded thermal images of vertical combustion of unmodified samples and those containing the maximum filler-content tested are shown in Figure 11. Those thermal images were recorded immediately after the burner was moved away. In the case of PBS plastic without filler, the flame is hardly thermally visible, and disappears completely after less than 30 s (Table 2), while PE burns completely. Flaming drops of molten plastic were observed dripping for both unmodified materials. The introduction of 50% filler in both cases caused the samples to burn completely, at a much higher flame. The temperature in the flame for both unfilled and filled material was higher during the burning of PE. Falling, flaming drops of melt plastic were also present in polyethylene containing 50% filler, while in the case of PBS50 they were not observed, but partially charred larger fragments of the material broke off less frequently and caused the cotton batting to ignite, which leads to the conclusion that this material generates less of a fire hazard than PE.

**Figure 12:**
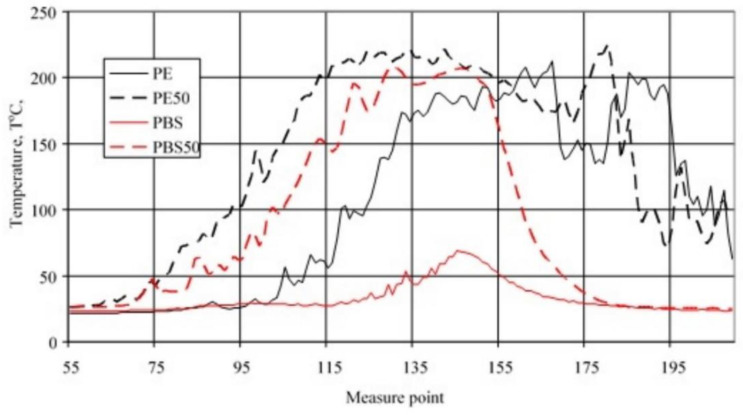
Temperature along the vertical axis of burning samples of unmodified PE and PBS (PE, PBS) and those containing 50% filler content (PE50, PBS50).

Figure 12 summarizes curves showing the temperature distribution along the vertical axis of burning PE and PBS samples, both for unmodified samples and those containing 50% filler. In the case of unmodified PBS, both the temperature value and the length of the area of its elevated values are significantly smaller than in the case of the other tested materials. This indirectly demonstrates the relatively small area occupied by the flame. The introduction of the filler into the PBS resulted in a significant increase in flame temperature and an increase in flame area. The curves obtained for PE indicate that at the time their values were recorded, the flame on the unmodified PE was already much closer to the holder than on the modified one, which may indirectly indicate a higher linear burning rate. However, the introduction of the filler increased the flame temperature.

### 3.5. Chemical Structure of Sample Residues after Flammability Test

**Figure 13:**
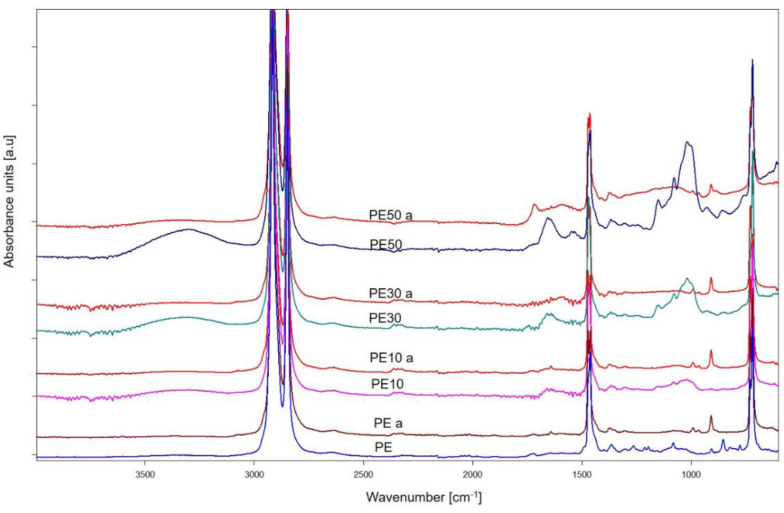
Spectra of samples made of polyethylene and its composition with bran, before and after the flammability test (a).

To gain insight into the chemical structure of residues of PE, PBS and their compositions after the burning test, the ATR–FTIR analysis was performed. Spectra of PE samples before and after burning are presented in Figure 13. Absorption bands at 2916 cm^−1^, 2849 cm^−1^, 1471 cm^−1^ and 719 cm^−1^ from stretching and deformation vibrations of methylene groups are present on the spectra of PE and its bran composition before the flammability test. The remaining absorption bands on the PE10, 30 and 50 spectra come from vibrations of the structural elements that make up the bran, including C=O, C-N, C-O-C, and -OH groups [44]. After the flammability test, the residue spectra still retain bands originating from the vibration of methylene groups, which indicates that polyethylene chains remained in the structure of the non-carbonized residue after combustion. In addition, a new low-intensity band at 991 cm^−1^ (vibrations of ether groups) and a band at 908 cm^−1^ from vibrations of unsaturated =CH_2_ groups appear. PE becomes thermally degraded in accordance with the depolymerisation mechanism, so the combustion residue may presumably contain short-chain PE. Changes are observed in the spectra after the flammability test in the bands originating from the bran. The bands in the range of 3600–3100 cm^−1^ (vibration of -OH groups), 1800–1600 cm^−1^ (vibration of carbonyl groups, among others) and in the range of 1200–950 cm^−1^ (vibration of C-O-C groups) disappear, which indicates thermal degradation of the organic biofiller.

**Figure 14:**
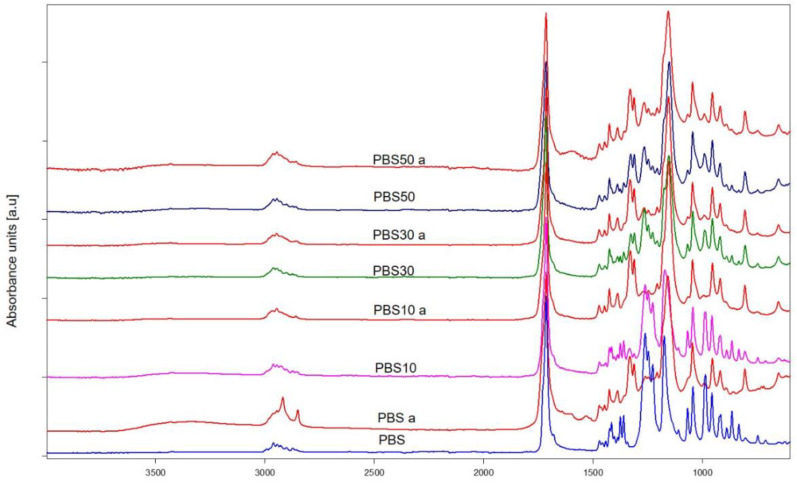
Spectra of samples made of poly(butylene succinate) and its composition with bran, before and after the flammability test (a).

FTIR spectra of the composition with PBS before and after the flammability test are shown in Figure 14. PBS belongs to the polyester group, while bran is a lignocellulosic material. The spectra of pure PBS, as well as the composition containing bran before and after the flammability test, show changes mainly in the vibration region of the ester groups. Except for PBS50, for all samples after burning, the intensity of the vibration absorption band of the carbonyl group (1714 cm^−1^) decreased at the expense of an increase in the intensity of the vibration absorption band of the C-O-C group (1174 cm^−1^/1154 cm^−1^). In addition, the C-O-C vibration band has shifted from 1174 cm^−1^ to 1154 cm^−1^. This proves that during combustion ester bonds decomposed and C-OH groups were formed, emerging into the structure of alcohols and possibly carboxylic acids. The disappearance of absorption bands is observed on the spectra recorded after the flammability test in the range 1264–1227 cm^−1^, which originate from C-O-C symmetric vibrations in the esters, and for PBS50 this band has a lower intensity than before the test. Moreover, a band at 1600 cm^−1^ is present only on the spectra of PBS50a and PBSa, which can be attributed to the vibration of unsaturated C=C groups. In addition, an increase in the intensity of the absorption bands at 1332 and 1312 cm^−1^ is clearly visible on the spectra of the flammability-test residues of the formulation with PBS, while in the case of PBS these bands were not present in the spectrum before the flammability test. These spectra can be attributed to vibrations of structural fragments with the nature of carboxylic acids. Interestingly, for PBS after the flammability test, the spectral pattern changes within the vibration of the methyl and methylene groups (2990–2800 cm^−1^). The two absorption bands from the vibration of -CH_2_- groups at 2918 and 2850 cm^−1^ may be due to the formation of long-chain hydrocarbon groups on the surface of the sample under study. This effect is not as pronounced for compositions with bran.

## 4. Conclusions

Biodegradable poly(butylene succinate) (PBS) has significantly lower flammability compared with polyethylene (PE). During horizontal flammability tests of (PBS) samples, a lower flame-travel rate and lower values of maximum temperature in the longitudinal axis were observed, compared with the tested PE samples. During the vertical burning of PBS, the flame disappeared completely after less than 30 s, while the PE samples were burned completely.

In the case of polyethylene (PE), the introduction of bran into the composition resulted in an increase in the linear rate of flame travel. With the horizontal location of the sample, the largest increase was observed at 30%wt of bran content. With the content at 50%wt, a favourable increase in burning time and a decrease in burning rate were observed, which is characteristic of highly filled polyolefin plastics. In vertically located PE samples, a successive reduction of their burning time was observed as the bran content increased.

The introduction of bran caused a successive increase in the linear burning rate of the PBS samples. As in the case of PE, a decrease in the value of this value was observed at 50% wt bran content. It should be noted, however, that the burning rate values are significantly lower, compared with the PE matrix compositions. 

In the vertical flammability test, the introduction of 10%wt bran into PBS resulted in longer burning times, but at the higher bran contents of 30%wt and 50%wt, a complete burning of the samples was observed.

In conclusion, it can be said that poly(butylene succinate) (PBS) alone, as well as its compositions with wheat bran, pose a lower fire hazard compared with polyethylene (PE) and its compositions with bran.

FTIR studies of the chemical structure of the uncarbonized polyethylene (PE) flammability-test residue indicate the presence of a PE-structured polymer, while the disappearance of bands originating from the biofiller on the spectra of the PE-matrix compositions indicates its almost complete oxidation during combustion. In the case of residues from the PBS flammability-test and its bran composition, FTIR analysis revealed the presence of carboxyl and alcohol groups in their structure, resulting from the degradation of ester bonds in the polyester. The residue of pure PBS additionally revealed the presence of long-chain hydrocarbon structures.

The tested biocomposites without the use of flame retardants cannot be used in applications exposed to flame and in places with an increased risk of fire hazard. Potential reduction of the flammability of the tested compositions without losing their ecological properties, especially in the case of compositions based on PBS, can be obtained by introducing, for example, a melamine-based flame retardant.

## Figures and Tables

**Table 1:** Experimental design.

Experimental Design Layout	u, %wt	Matrix Material
PE	0	PE
PE10	10	PE
PE30	30	PE
PE50	50	PE
PBS	0	PBS
PBS10	10	PBS
PBS30	30	PBS
PBS50	50	PBS

**Table 2:** Results of horizontal flammability tests.

Experimental Design Layout	Burning Time of Measuring Length t_1_, s	Linear Combustion Rate *v*, mm/min	Flammability Class According to Horizontal Test
PE	157.64	28.55	HB40
PE10	128.33	35.07	HB40
PE30	98.27	45.79	HB75
PE50	141.02	31.91	HB40
PBS	264.76	17.00	HB40
PBS10	203.78	22.08	HB40
PBS30	129.00	34.88	HB40
PBS50	141.67	31.76	HB40

**Table 3:** Results of vertical flammability tests.

Experimental Design Layout	Burning Time after First Ignition t_2_, s	Sample Burned Completely YES or NO	Ignition of the Cotton Batting YES or NO
PE	78.29	YES	YES
PE10	40.40	YES	YES
PE30	38.44	YES	YES
PE50	30.90	YES	YES
PBS	28.89	NO	YES
PBS10	28.72	NO	YES
PBS30	50.84	YES	YES
PBS50	41.85	YES	YES

## Data Availability

The data presented in this study are available on request from the corresponding author.
